# Inbreeding in *Mimulus guttatus* Reduces Visitation by Bumble Bee Pollinators

**DOI:** 10.1371/journal.pone.0101463

**Published:** 2014-07-18

**Authors:** David E. Carr, T’ai H. Roulston, Haley Hart

**Affiliations:** 1 Blandy Experimental Farm, University of Virginia, Boyce, Virginia, United States of America; 2 Southeastern High School, Detroit, Michigan, United States of America; Central China Normal University, China

## Abstract

Inbreeding in plants typically reduces individual fitness but may also alter ecological interactions. This study examined the effect of inbreeding in the mixed-mating annual *Mimulus guttatus* on visitation by pollinators (*Bombus impatiens*) in greenhouse experiments. Previous studies of *M. guttatus* have shown that inbreeding reduced corolla size, flower number, and pollen quantity and quality. Using controlled crosses, we produced inbred and outbred families from three different *M. guttatus* populations. We recorded the plant genotypes that bees visited and the number of flowers probed per visit. In our first experiment, bees were 31% more likely to visit outbred plants than those selfed for one generation and 43% more likely to visit outbred plants than those selfed for two generations. Inbreeding had only a small effect on the number of flowers probed once bees arrived at a genotype. These differences were explained partially by differences in mean floral display and mean flower size, but even when these variables were controlled statistically, the effect of inbreeding remained large and significant. In a second experiment we quantified pollen viability from inbred and self plants. Bees were 37–54% more likely to visit outbred plants, depending on the population, even when controlling for floral display size. Pollen viability proved to be as important as floral display in predicting pollinator visitation in one population, but the overall explanatory power of a multiple regression model was weak. Our data suggested that bees use cues in addition to display size, flower size, and pollen reward quality in their discrimination of inbred plants. Discrimination against inbred plants could have effects on plant fitness and thereby reinforce selection for outcrossing. Inbreeding in plant populations could also reduce resource quality for pollinators, potentially resulting in negative effects on pollinator populations.

## Introduction

The detrimental effects of inbreeding in naturally outbreeding plant populations have been extensively documented (e.g., [1]–[3]). Most of these studies have focused on physiological, morphological, or individual fitness traits, but recent studies have documented negative effects of inbreeding in plants on their interactions with herbivores (reviewed in [4]) and pathogens [5]–[7]. These studies have demonstrated that inbreeding can have not only population-level effects but may have community-level effects as well. This study will address the effect of inbreeding on plant-pollinator interactions in experimentally inbred populations of *Mimulus guttatus*.

Inbreeding has been demonstrated to negatively affect traits potentially important to attracting pollinators in *M. guttatus*. Inbred *M. guttatus* produce smaller corollas [8], [9] and fewer flowers (e.g., [10], [11]) relative to outbred plants, altering potentially important visual cues to incoming pollinators. Inbreeding also reduces pollen production and viability in *M. guttatus* [10], [12], [13], [14], reducing the quantity and quality of a potentially important reward for pollinators. Robertson *et al*. [15] demonstrated that British bumble bees (*Bombus* spp.) can, in fact, discriminate among *M. guttatus* genotypes varying in pollen quality and quantity. If pollinators respond similarly to inbred and outbred genotypes, the competitive ability and ultimately the fitness of inbred plants may be greatly impaired. Furthermore, an elevated level of inbreeding in a plant population might consequently result in a substantially degraded resource for pollinators.

Although a number of studies have examined the effect of pollinator service on selfing rates in plant populations [16], only one study has examined the effects of inbreeding on pollinator service. Ivey & Carr [9] found that a natural community of pollinators visited experimentally inbred plants 34% less often when given a choice between inbred and outbred *M. guttatus*. Handling time also increased by 49% for pollinators visiting inbred plants. Syrphid flies were by far the most common floral visitors (67.4%), although the response to inbred and outbred plants was similar for both syrphids and large bees (*Bombus* and *Apis*). Although this study controlled for both floral display and corolla size variation, it did not examine the influence of floral rewards on pollinator behaviour.

This study will focus on a single species of pollinator, *Bombus impatiens*, and its response to experimentally inbred populations of *M. guttatus*. Bumble bees are the primary pollinators of *M. guttatus* in both its native [17], [18] and introduced [15] range. The experiments were conducted in the greenhouse where environmental variables and, especially, resource levels could be more closely controlled. In examining the response of pollinators to inbreeding in *M. guttatus*, we asked the following questions: 1) Does inbreeding reduce the probability that a bumble bee will visit a plant? 2) Does inbreeding reduce the number of flowers visited once a bee arrives at a plant? To understand what might be driving pollinator response, we further asked: 3) Do inbreeding-induced changes in display traits account for the differences in visitation? 4) Does pollen viability account for the variation in visitation rates by bumble bees? We found that bumble bees strongly discriminated against inbred plants independent of display traits. The pollen reward may have played a role in bee choice, but pollen viability explained only a small amount of variation.

## Materials and Methods

These experiments were conducted at the University of Virginia's Blandy Experimental Farm with the permission of its Director. No permits were required. *Mimulus guttatus* seed was collected from populations at the edge of public roads. No specific permissions were required for these locations/activities. Field studies did not involve endangered or protected species. Coordinates for the three populations sampled in this study were as follows: Santa Clara County, CA (37° 17′ N, 122° 09′ W), Napa County, CA (38° 33′ N, 122° 22′ W), and Tuolumne County, CA (37° 50′ N, 120° 28′ W). The raw data from Experiment 1 and 2 are available in Data S1.

### Study Species

*Mimulus guttatus* DC (Phrymaceae, [19]) ranges throughout western North America from Mexico to Alaska, occupying a variety of moist, open habitats. It is capable of producing 100 or more large (∼20–30 mm wide), yellow, hermaphroditic flowers, each capable of producing hundreds of seeds. Populations are typically annual, but in environments that remain wet year round, perennial forms can occur [20]. *Mimulus guttatus* typically exhibits a mixed mating system with estimates of outcrossing rates across populations varying from about 75% selfing (*t* = 0.25) to complete outcrossing (*t* = 1.0), averaging *t* ≈ 0.60 [21]–[23]. Bumble bees (*Bombus spp*.) are known to be an important pollinator [17], [18], [24], [25], though syrphid flies and smaller bees are also frequent visitors [9], [26].

### Experiment 1: Effects of inbreeding on bumble bee visitation

In May 1997 seeds from a large, annual population of *M. guttatus* in Santa Clara County, CA were collected. In fall 1998, one plant from each of 20 field-collected maternal families was used for controlled pollinations. On each plant we emasculated flowers in bud one to two days prior to anthesis. After anthesis, outcross pollinations were accomplished by rubbing anthers from a randomly chosen pollen donor onto the receptive stigma of one of the emasculated flowers on the pollen recipient. Plants were used as pollen donors only once for outcross pollinations. Self-pollinations were accomplished by rubbing anthers containing self pollen (collected from a flower on the same plant) onto the stigma of the other emasculated flower. In spring 1999, one plant was grown from the self seed (Self1) from each maternal family. These plants were self-pollinated as described above to produce seed (Self2) that had now been selfed for two generations.

*Bombus impatiens* is a medium-sized, generalist bumble bee native to the eastern United States. This species has been shown to make foraging decisions based on flower age [27], flower color and pattern [28]–[31] and can use color cues to adjust their foraging behavior on flowers that have different reward mechanisms [32]. The *B. impatiens* used in this study were obtained through Koppert Biological Systems, USA. This artificial hive allowed us to control the number of bees foraging at any given time. The bees were not permitted to forage in the greenhouse outside of our observation periods. We provisioned the hive with ground pollen pellets taken from honey bees and a 40% sucrose solution *ad lib* to supplement resources gained during foraging trials.

**Figure 1:**
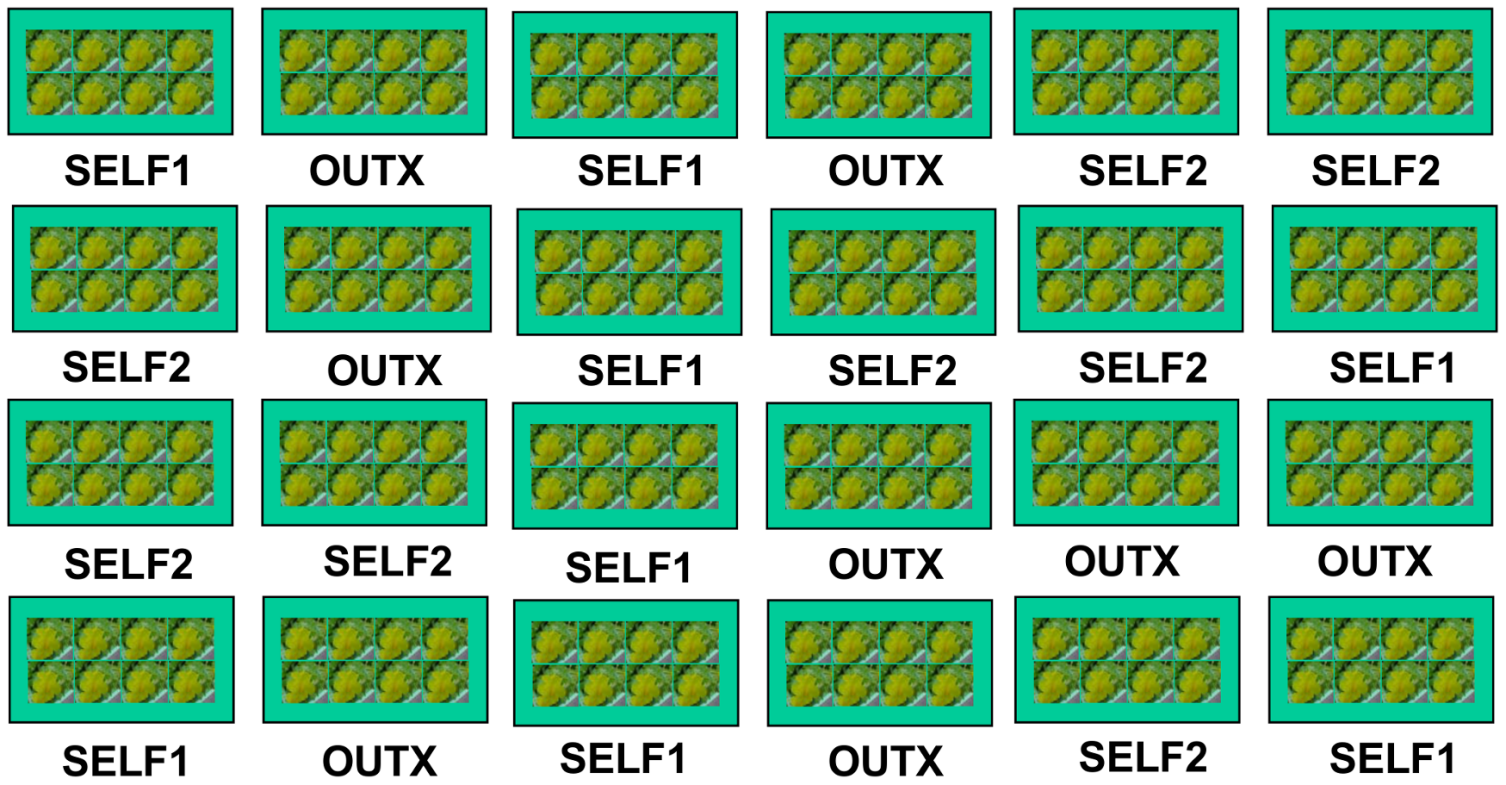
The layout of octets on a single greenhouse bench for Experiment 1. Eight inbred (SELF1 and SELF2) or outbred (OUTX) plants from a single maternal family were arranged in a single tray (an “octet”). Twenty-four octets (eight Self1, Self2, and outbred) were randomly arranged on each of the five greenhouse benches.

On 8 December 2001, inbred (Self1 and Self2) and outbred seeds from each maternal family were sown into 72 mm square pots. On 21 December 16 seedlings from each maternal family x inbreeding treatment combination (hereafter referred to as “genotypes”) were transplanted into their own 72 mm pots. Eight plants of like genotypes were arranged in the center of a 55×28 cm tray. These “octets” were constructed to ensure that fresh flowers of each genotype were available to bees on any given day. Octets from four maternal families (2 replicates each of outbred, Self1, and Self2) were arranged in random positions on each of 5 greenhouse benches (Fig. 1). On each table, therefore, bees had the choice of eight outbred octets, eight Self1 octets, and eight Self2 octets. The arrangement of trays on benches isolated octets into distinct “foraging patches” separated by 30 cm within rows and 16 cm between rows.

We wanted to make our pollinator observations during a period of relatively high resource availability in order to provide the foragers with as many options as possible throughout the trial. In order to ensure high resource availability at the start of each observation session, three greenhouse benches (in addition to the five focal benches) were filled with *M. guttatus* to provide more resources for the foragers so that they were not forced to forage only on our experimental plants. Preliminary observations suggested that an unrestricted number of foragers from the hive were capable of severely depleting resources in the greenhouse after about 2 hours (by this time we observed many bees opening flower buds to extract pollen from their undehisced anthers). During our actual observations we limited the number of foraging bees to no more than 12 at any one time, and observation periods were restricted to 1 hour and 15 minutes. Bees were not permitted access to the greenhouse after the end of each daily observation period.

Foraging observations were made during the morning hours from 8 February to 1 March 2002. At the beginning of any observation day, one observer would randomly choose a greenhouse bench at which to start. The other observer would start at a bench at least one bench removed from the first observer. At the end of a 15 minute observation period, the two observers would rotate to the next bench. The observation day would end once both researchers had visited each of the five benches.

For each period, observations would start with the arrival of a bee at the bench. This focal bee would be observed as it moved from flower to flower until it left the bench. The genotype of each octet would be recorded as would the number of flowers visited at each octet. We defined a visit as a bee landing on the labellum of the flower and inserting at least its head into the corolla tube. Once the focal bee left the table, the observer would wait until another bee arrived. This new focal bee would be followed as before. If the observer was following a bee after the 15 minute period expired, he would continue making observations until that bee left the table so that its full set of choices could be recorded.

Expecting that visual cues may strongly influence pollinator visitation, the total display size (number of flowers) of each octet was determined on each observation day. We were unable to collect display size data on 26 February, and results from this day were omitted in analyses that required display data. Corolla widths were measured on a single flower on each plant of an octet early in the experiment (8–13 February).

#### Analyses: Experiment 1

In our analyses of bee visitation, we treated our observations as repeated measures with individual octets as “subjects” and observation date as the time element. Maternal family was treated as a random effect, and the degree of inbreeding (outbred, Self1, and Self2) was analyzed as a fixed effect. All analyses were performed using SAS Proc Mixed [33] using the “repeated” statement with the covariance structure selected based on lowest AIC. Random effects were evaluated by log-likelihood ratio tests.

In the first repeated measures analysis, we examined the total number of visits to each octet during an observation period as the dependent variable (a bee arriving at an octet was regarded as a single visit regardless of the number of flowers it sampled before leaving the octet). In a second analysis we examined the mean number of flowers visited per arrival at individual octets as our dependent variable. In both analyses the dependent variable was log-transformed to conform to ANOVA assumptions.

Because floral display size (the number of flowers open on any given day) was found to have a positive correlation with both visitation to octets and the number of flowers probed per visit, it was used as a covariate in separate repeated measures ANCOVA analyses (square-root transformed). A preliminary heterogeneity of slopes model indicated that the homogeneity of slopes assumptions held for the display size covariate.

We could not use corolla width as a covariate in a repeated measures analysis as we did above for display size because corolla width was measured only once during the experiment. However, we were able to use it in an analysis of covariance with the total number of visits (summed across the entire experiment) as the dependent variable. In this model we examined the effect of inbreeding level (fixed effect), maternal family (random effect), and their 2-way interaction (random effect). We included both corolla width and total flower production as covariates. These two variables were uncorrelated (*r* = 0.049, *p* = 0.63) and therefore could independently control for variation due to floral display traits. Total flower production was square-root transformed. A preliminary heterogeneity of slopes model indicated that the homogeneity of slopes assumptions held for the corolla width covariate.

Finally, we also conducted a multiple regression with corolla width and total flower production as independent variables and the number of visits to each octet as the dependent variable in order to determine the relative contributions of these traits. The best fit model was evaluated by lowest AIC.

### Experiment 2: Effects of inbreeding and pollen viability on visitation

In the summer of 2007 we conducted a second experiment in order to test for the effect of reward quality on pollinator visitation. We used two populations of *M. guttatus*, M13 from Pope Valley, Napa County CA and DP from the Don Pedro Reservoir, Tuolumne County, CA. In a greenhouse at Blandy Experimental Farm, field collected seed from each population was randomly crossed within a population for one generation. Randomly selected plants from each population were then selfed for one generation and randomly outbred to create 28 and 29 maternal families of inbred and outbred plants from populations DP and M13, respectively. Crossing techniques and growing conditions were identical to those described in Experiment 1.

Five seedlings from each level of inbreeding from each maternal family were transplanted and randomized within five blocks (*n* = 300 plants per population), 10 plants to a tray to allow about 7 cm of space between plants. Plants from M13 and DP were housed on separate greenhouse benches that accommodated all five blocks. Extra (non-focal) plants were added to the greenhouse to increase resource levels as before. We collected anthers from two freshly opened flowers at the second and third node from each experimental plant and placed them in a microcetrifuge tube with lactophenol in analine blue to stain viable pollen grains [34]. After 10 minutes of sonication, we examined a 15 µl drop of each sample under a compound light microscope and counted approximately 100 pollen grains, scoring darkly stained grains as viable and unstained grains as inviable. Two replicate pollen counts were made for each sample, and we calculated a mean proportion of viable pollen across the replicates for each individual plant.

Visitation to these plants was monitored for six days. Prior to each observation period, we counted the number of open flowers on each plant. Each population was monitored by a single observer, with the observers alternating between populations each day. Each observer followed a single bee as it arrived at a greenhouse bench (i.e., a *M. guttatus* population) and recorded each individual plant visited and the number of flowers probed per visit. Observation periods lasted 45–60 minutes, depending on the level of bee activity.

#### Analyses: Experiment 2

Differences between inbred and outbred plants in pollen viability were analyzed with a generalized linear model using SAS Proc Glimmix and a logit link function. Inbreeding level was treated as a fixed effect, and block, maternal family, and a family × inbreeding interaction were included in the model as random effects. Each population was analyzed separately.

Visitation and floral probes were analyzed by repeated measured ANCOVA using SAS Proc Mixed. Each individual plant was designated as the “subject” in the analysis. Inbreeding, trial date, and their interactions were treated as fixed effects. Block, family, and a family × breeding interaction were treated as random effects. Daily floral display size was used as a covariate. The variance-covariance structure was chosen based on AIC. Each population was analyzed separately.

Finally we employed a multiple regression to evaluate the effects of pollen viability and mean floral display size (averaged across the 6 trial dates) on mean visitation (averaged across the 6 trial dates). Visitation was log-transformed. The best fit model was evaluated by lowest AIC.

## Results

### Experiment 1

Observations were made on a total of 18 days during the course of the experiment. This amounted to a total of 41-person hours of observation time and a total of 5021 visits to individual octets and 13,064 individual floral probes.

**Figure 2:**
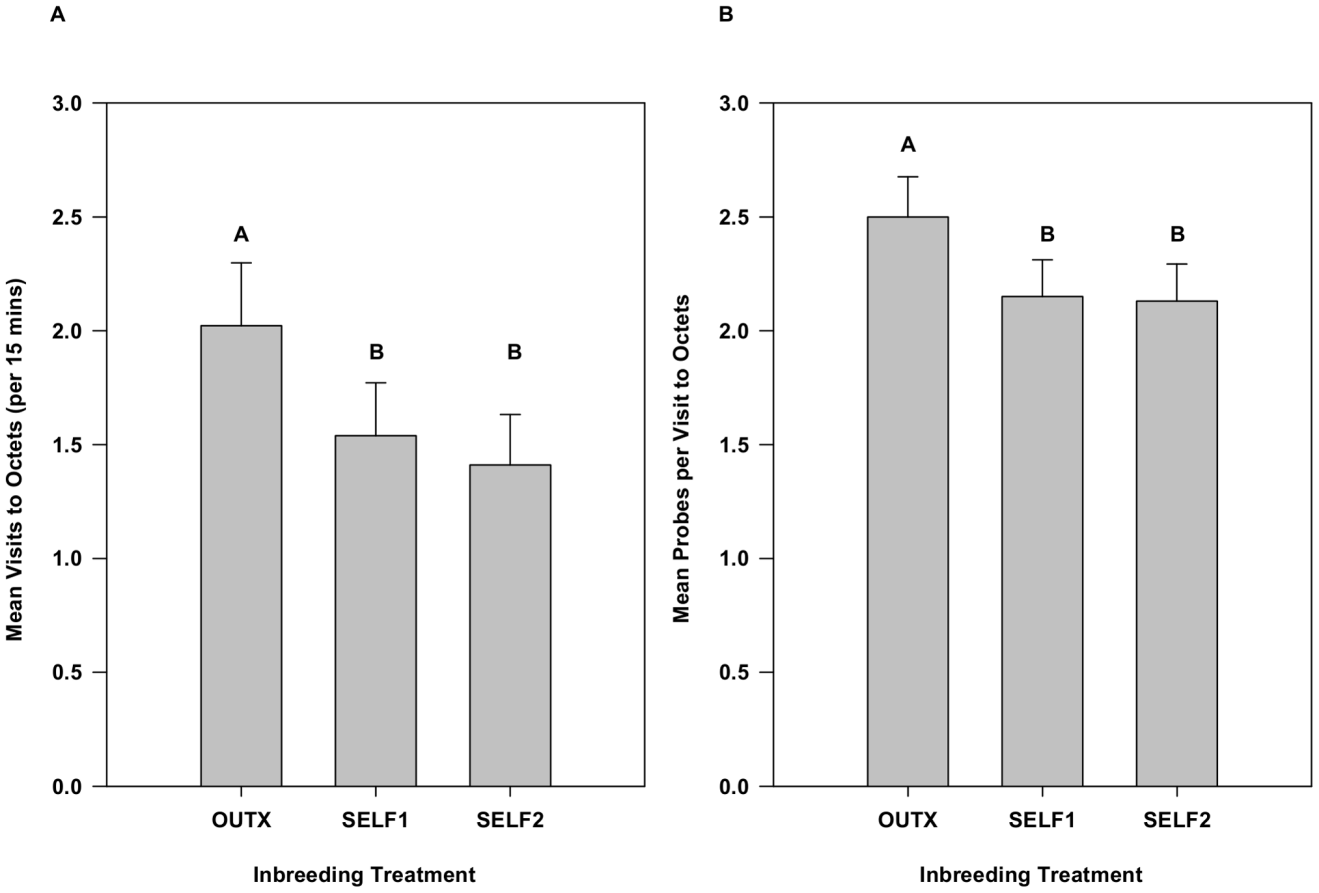
The effect of inbreeding on A) bee visitation to octets and B) probes into flowers for Experiment 1. Means represent least squares means from repeated measures ANOVAs using SAS proc mixed. Inbreeding reduced visitation and probing by bumble bees. Means with different letters are significantly different from one another using Tukey-Kramer multiple comparison test. Error bars indicate upper 95% confidence limits.

Repeated measures ANOVA revealed that bees visited significantly more outbred octets (*F*_2,38_ = 8.90, *p* = 0.0007) than either of the inbred octets (Fig. 2a). Outbred octets received 31% more visits than Self1 and 43% more visits than Self2. Visitation at the two inbred octets did not differ significantly from one another based on a Tukey-Kramer test. A significant inbreeding×family interaction indicated that the effect of inbreeding on bee visitation varied across maternal families (χ^2^ = 45.5, *p*<0.0001). For half of the families, visitation declined continuously as they became increasingly inbred, but seven families showed higher visitation to the Self2 plants relative to Self1.

When bees arrived, they probed significantly more flowers at outbred octets (*F*_2,38_ = 9.13, *p* = 0.0006) than either of the inbred octets (Fig. 2b). The differences are small in absolute magnitude, amounting to no more than 0.35 flowers per octet visit on average, but bees visited 16% and 17% more flowers in outbred octets relative to Self1 and Self2, respectively. The inbred plants did not differ significantly from one another. The effect of the inbreeding treatments on the number of flowers probed by bees varied significantly among plant families (χ^2^ = 5.1, *p* = 0.012), but in 14 of the 20 families, bees probed more flowers on outbred plants than both Self1 and Self2.

Inbreeding did not have an overall effect on the mean number of open flowers (i.e., display size), with daily means of the three groups ranging from 22.3 – 24.9 flowers per octet (*F*_2,38_ = 0.78, *p* = 0.4883). There was, however, a significant inbreeding×day interaction (*F*_34,2030_ = 2.93, *p*<0.0001). During the first nine days of observation, outbred octets typically had larger mean display sizes than inbred octets, but inbred octets averaged larger display sizes thereafter.

**Figure 3:**
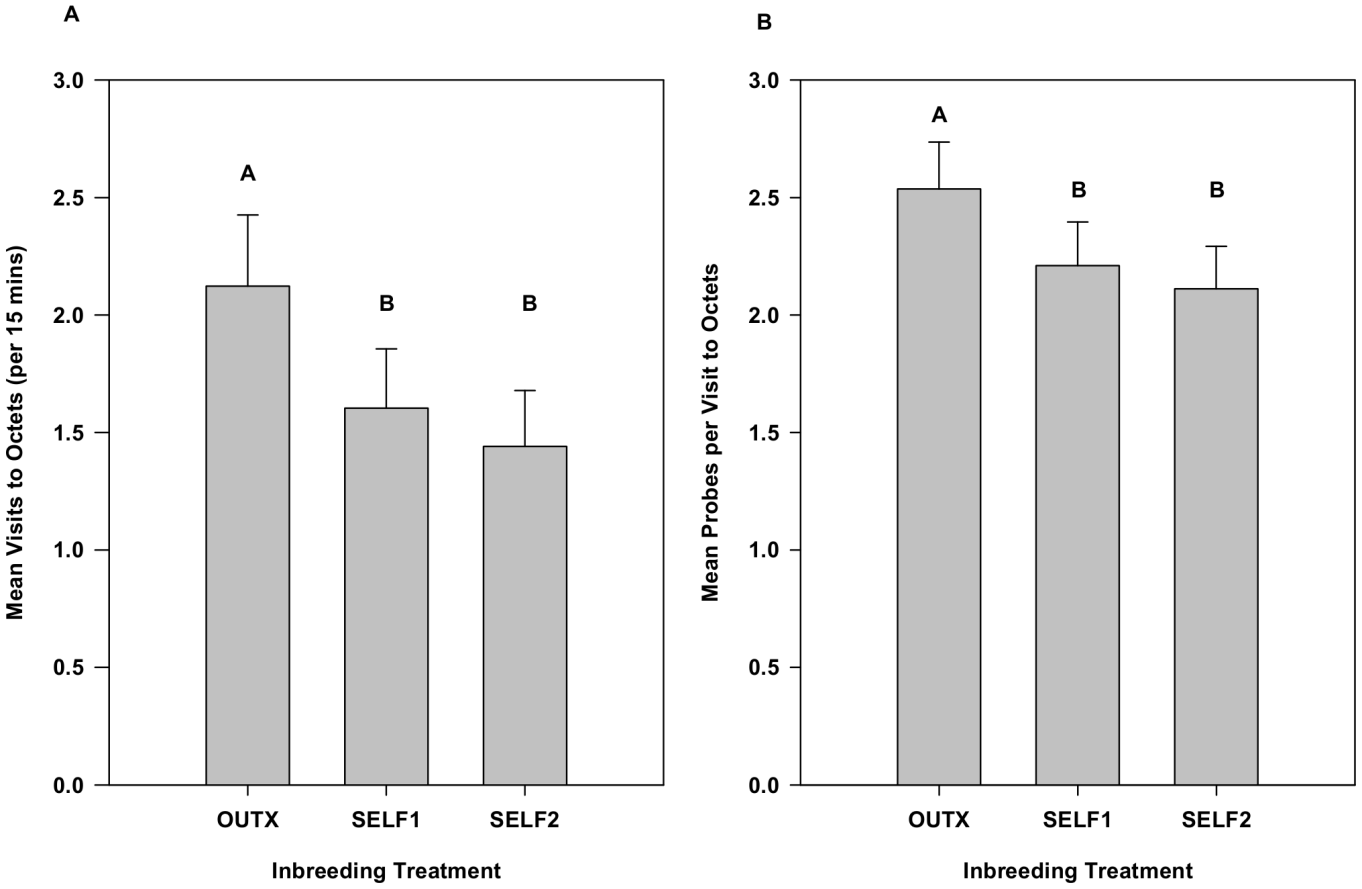
The effect of inbreeding while controlling for floral display size on A) bee visitation to octets and B) probes into flowers for Experiment 1. Means represent least squares means from repeated measures ANCOVAs using SAS proc mixed after the effect of display size had been removed. Although display size had a strong effect on both response variables, inbreeding effects on bumble bee behaviour persisted in these ANCOVAs. Means with different letters are significantly different from one another using Tukey-Kramer multiple comparison test. Error bars indicate upper 95% confidence limits.

Because of the differences in flower production over the course of the experiment and because daily flower display would likely influence visitation, we used this variable as a covariate in examining visitation. The effect of flower display size was indeed significant (*F*_1,2030_ = 38.46, *p*<0.0001), but even when controlling for display size, a strong inbreeding effect remained (*F*_2,38_ = 9.41, *p* = 0.0005). The relative differences between treatments in the ANCOVA were nearly the same as the analysis without the covariate (Fig. 3a). Controlling for floral display, mean visitation to outbred octets was significantly greater than each of the two inbred octets (32% and 47% greater relative than Self1 and Self2, respectively), but the inbred octets did not differ significantly from one another.

Similarly, a repeated measures ANCOVA found differences among inbreeding treatments in the mean number of flowers probed once bees visited octets (*F*_2,38_ = 10.05, *p* = 0.0003) even after controlling for the significant effect of the covariate, display size (*F*_1,1598_ = 66.69, *p*<0.0001). The effect sizes were similar to those seen without the covariate, with outbred octets receiving 15% and 20% fewer probes per visit relative to Self1 and Self2, respectively (Fig. 3b).

Corolla widths differed significantly among the levels of inbreeding (*F*_2,38_ = 4.50, *p* = 0.0177). Corollas on outbred plants were significantly larger (8%) than corollas on inbred plants (outbred mean = 22.94 mm versus 21.23 mm and 21.25 mm for Self1 and Self2, respectively), but mean corolla width did not differ significantly between inbred plants. A significant inbreeding×family interaction indicated that corolla width varied across maternal families in its response to inbreeding, however (χ^2^ = 26.0, *p*<0.0001).

**Figure 4:**
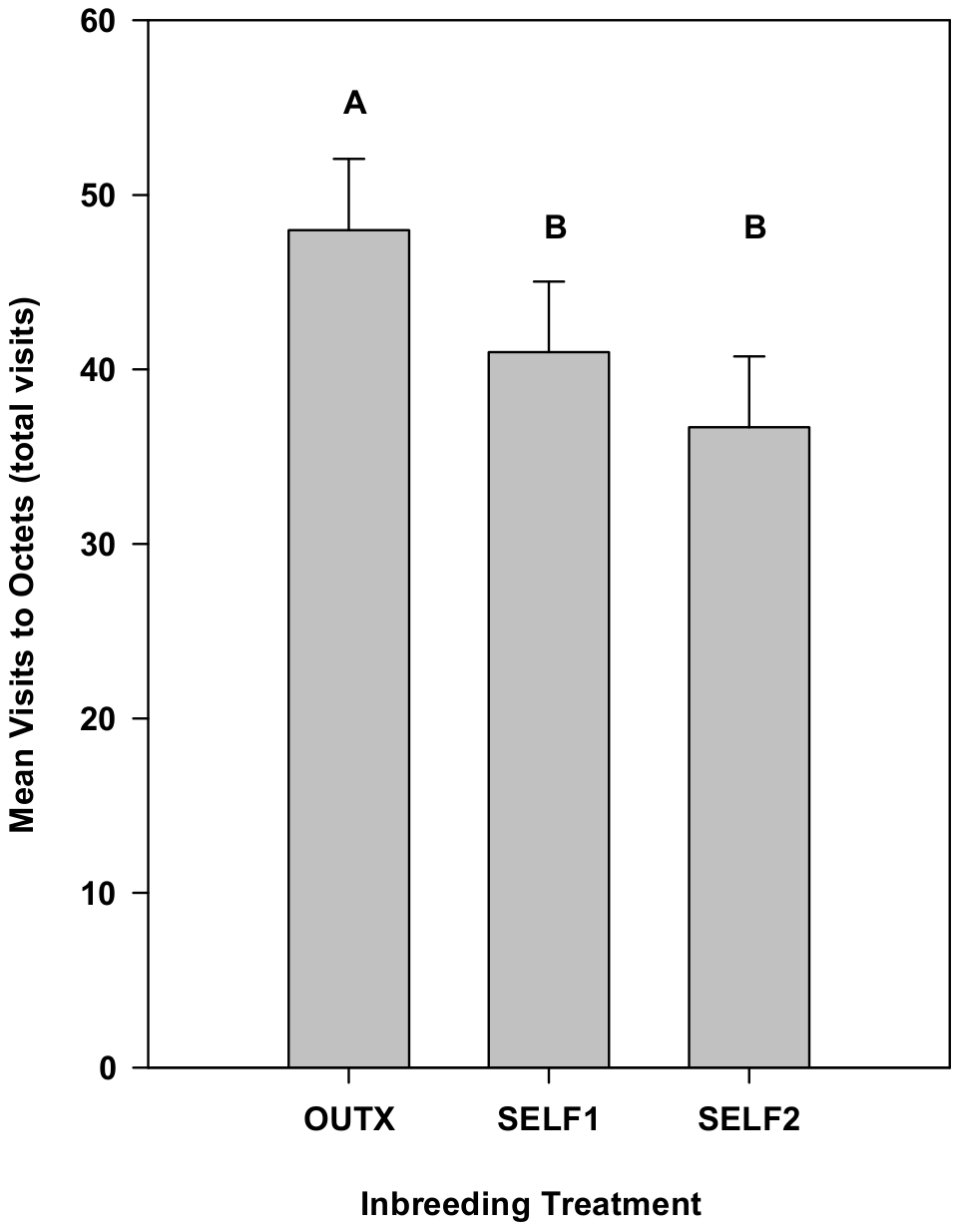
The effect of inbreeding on the total number of bee visits to octets while controlling for mean floral display size and mean corolla width for the duration of Experiment 1. Means represent least squares means from repeated measures ANCOVAs using SAS proc mixed after the effect of display size and corolla width had been removed. After statistically controlling for these two important visual cues, bumble bees still discriminate against inbred plants. Means with different letters are significantly different from one another using Tukey-Kramer multiple comparison test. Error bars indicate upper 95% confidence limits.

In an analysis of the total number of visits to each octet (summed across the entire experiment) using both corolla width and mean flower production as covariates, there was still a significant inbreeding effect on the number of *Bombus* visits (*F*_2,38_ = 7.79, *p* = 0.0015), although the effect of inbreeding was relatively smaller than in the previous analyses (Fig 4). Controlling for these covariates, visitation to outbred octets was significantly greater than both Self1 (17% higher) and Self2 (31% higher), but the inbred groups did not differ significantly from one another. Again, the response of bees to inbreeding varied across maternal families (χ^2^ = 52.1, *p*<0.0001). Both covariates explained a significant amount of variation in the number of *Bombus* visits per octet (*F*_1,57_ = 35.40, *p*<0.0001 and *F*_1,57_ = 5.40, *p* = 0.0237 for corolla width and total flower production, respectively).

In a multiple regression analysis with corolla width and mean flower production as independent variables, the best supported model included both variables (ΔAIC = 9.3 relative to a model with only corolla width). Corolla width explained a higher proportion of the overall variation in the total number of *Bombus* visits to octets than did total flower production (*R* ^2^ = 0.37, versus *R* ^2^ = 0.07, respectively) and had a higher standardized regression coefficient (*b_cw_* = 0.59 versus *b_flowers_* = 0.27). This was so despite the fact that the coefficient of variation for flower production (cv = 33%) was more than twice as great as that for corolla width (cv = 15%).

### Experiment 2

**Figure 5:**
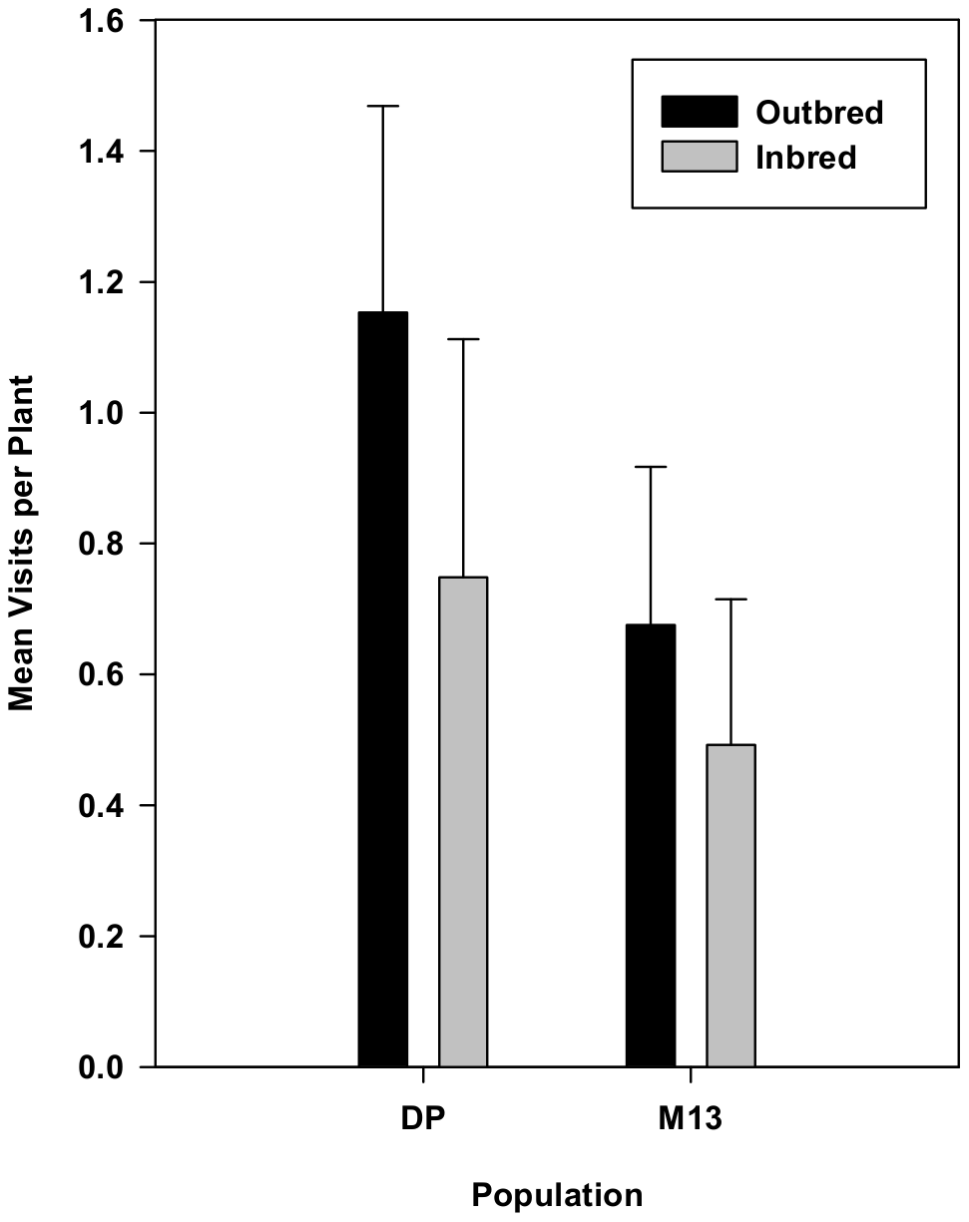
The effect of inbreeding on bee visitation to two populations of plants (DP and M13) in Experiment 2 while controlling for floral display size. Means represent least squares means from repeated measures ANCOVAs using SAS proc mixed after the effect of display size had been removed. In both populations, bumble bees visited inbred plants significantly less often. Error bars indicate upper 95% confidence limits.

We observed a total of 856 visits to population DP plants and 999 visits to population M13 plants. Overall, bees were 54% more likely to visit outbred DP plants than inbred DP plants (Fig. 5; *F*_1,20_ = 9.52, *p* = 0.0058) in a repeated measures ANCOVA, using floral display size as a covariate. Bees were 37% more likely to visit M13 outbred plants (Fig. 5; *F*_1,20_ = 5.65, *p* = 0.0248), and this effect was consistent across dates. In both populations, the effect of floral display size was highly significant (*p*<0.0001).

Neither population showed a significant difference in the number of flowers probed once a bee arrived at a plant (*F*_1,16_ = 1.75, *p* = 0.2044 and *F*_1,24_ =  0.41, *p* = 0.5299 for populations DP and M13, respectively). In both populations, bees probed approximately 1.4–1.5 flowers per plant per arrival, on average, regardless of inbreeding treatment.

In both populations, outbred plants had significantly higher pollen viability than inbred plants, but the inbreeding effect was more pronounced in M13. Inbreeding reduced M13 pollen production by 23%, but by only 17% in DP (*F*_1,26_ = 47.45, *p*<0.0001; *F*_1,15_ = 12.90, *p* = 0.0027, respectively). The best supported multiple regression model for predicting average bee visitation to plants in DP included mean floral display as the only predictor variable (*R* ^2^ = 0.07). The next highest ranked model included both mean floral display and pollen viability and had a ΔAIC of only 0.68, but this more complex model had minimal improvement in explanatory power (*R* ^2^ = 0.08). The best supported model for population M13 included both mean floral display and pollen viability, and it was substantially better than the next best supported model (ΔAIC = 3.36), which included only pollen viability. The model had very modest explanatory power (*R* ^2^ = 0.08), but the standardized partial regression coefficient for pollen viability (0.16) was comparable to the coefficient for mean floral display size (0.22).

## Discussion

### Inbreeding and pollinator visitation

Inbreeding had a significant and substantial effect on the visitation rates of *Bombus impatiens* to *Mimulus guttatus* plants in these experimental greenhouse populations. In the first experiment, bees were 31% more likely to visit outbred octets than Self1 octets and 43% more likely to visit outbred octets relative to Self2. In the second experiment, bees were 37%–54% more likely to visit outbred plants than plants from one generation of selfing. These differences are comparable to the 34% reduction in visitation to inbred plants found in a controlled field study of *M. guttatus* in which the pollinator fauna was dominated by syrphid flies [9].

Inbreeding appeared to act primarily on the decisions bees made about where to forage rather than how long to forage once arriving. In experiment 1, inbreeding did affect the number of flowers probed once a bee arrived at an octet, but this effect was much smaller than the effect on arrival at octets. When a bee arrived at an octet, it typically visited about two flowers before moving to another octet. Although bees made as many as 25 floral probes at an octet, 77% of bees probed no more than three flowers per arrival. Ivey *&* Carr [9] found no difference between inbred and outbred plants in the number of flowers probed once pollinators arrived, and no differences were found here in experiment 2.

Our experiments allowed us to explore potential explanations for the observed discrimination against inbred plants by bumble bees. In both experiments, visitation was influenced, in part, by the size of the floral display and (at least in experiment 1) corolla size. These traits have been shown to be important in attracting bumble bees in studies of many other species (e.g., [35]–[38]). Both traits likely increase apparency, and they may convey information about the quantity of rewards. For example, corolla width has been found to be correlated with pollen production in *M. guttatus* [39]. Corolla size might also convey information about the cost of a visit if size affects handling time. Despite the importance of corolla size and floral displays in attracting bumble bees in our study, these two traits did not entirely explain the difference between visits to inbred and outbred octets. When the effects of floral display size and corolla size were controlled in analyses of covariance, the negative effects of inbreeding on visitation remained and the size of the effects were still substantial. Interestingly, in the second half of experiment 1, flowers on inbred plants outnumbered those on outbred plants, likely due to the effects of pollinator visitation on floral longevity [40], but bees still made more visits to outbred octets. This suggests that inbreeding reduces attractiveness beyond these obvious visual cues.

In experiment 2, inbreeding significantly reduced pollen viability, although the effect was stronger in population M13 than in DP. Pollen viability has been demonstrated to be positively correlated with protein content [41], indicating a potentially greater nutritional reward for visiting outbred plants. A multiple regression analysis indicated that in population M13, the best supported model for predicting pollinator visitation included both floral display size and pollen viability. The effect of each of these variables on pollinator visitation was essentially equivalent, based on their standardized regression coefficients. The total explanatory power of this model was weak, however (*R* ^2^ = 0.08), and pollen viability was not included in the best supported model for population DP (which showed substantially less variation in pollen viability).

The ability of bees to respond to variation in pollen rewards has been demonstrated in previous studies. Robertson *et al*. [15] presented data that showed that bumble bees preferred genotypes of *M. guttatus* that produced more abundant and higher quality pollen, but they showed little response to nectar variation. However, Wise et al. [42] found that bumblebees were equally likely to visit male-sterile and male-fertile *M. guttatus*. Despite the importance of varying nutrient levels in pollen [43], it is not clear if bees can directly assess cytoplasmic nutrients. A carefully controlled study using artificial pollen differing in nutrients found that honey bees could not detect variation in protein concentration, but clearly responded to differences in volatile compounds on the outside of pollen grains [44]. Other studies have shown that pollinators recognize and respond to anther/pollen volatiles, including choosing the sex [45] and the species [46] of flower to visit. It remains to be seen whether these known cues associated with host plant choice are affected by inbreeding in much the same way as pollen quantity and quality.

It seems very likely that other cues, unmeasured in this study, play an important role in pollinator foraging decisions as they interact with *M. guttatus*. Rae and Vamosi [47] have demonstrated that UV reflectance patterns are important in bumble bee responses to *M. guttatus*, and the production of volatile organic compounds (VOCs) appears to play an important role in bee-pollinated *Mimulus* species [48]. If these or other cues are affected by inbreeding, they could contribute to the pollinator discrimination against inbred plants that we observed. Inbreeding has been demonstrated to alter VOC emissions from *Solanum carolinense*, resulting in behavioural changes in ovipositing moths, herbivorous insects, and natural enemies [49], [50]. The effect of inbreeding on floral pigmentation in wild plants has not been explored.

### Evolutionary, ecological, and conservation implications

The reduced ability of inbred plants to attract pollinators could further reduce the fitness of inbred plants through pollen limited seed-set or by decreasing offspring quality through a proportionately greater contribution of autogamous self-pollination to seed-set [51]. If so, this could further reinforce selection for outcrossing. Greater attractiveness to pollinators could have negative effects on plant fitness, however, if this results in higher levels of geitonogamous pollination. Karron *et al*. [52] demonstrated that larger floral displays resulted in higher selfing rates in *M. ringens*, apparently due to greater within-plant foraging by *Bombus* on plants with the largest displays [38]. Ivey *&* Carr [9] found that selfing rates of outbred *M. guttatus* were significantly higher than in inbred plants, but any difference in geitonogamy is likely small since the number of flowers probed per visit by pollinators did not differ between inbreeding treatments in that study. The observed family variation in the response of pollinators to inbreeding could also affect mating-system evolution [53], [54], especially since we observed a number of families with higher visitation in Self2 plants relative to Self1.

The idea that genetic variation in one species can have effects on sympatric interacting species and even on ecosystem processes is gaining in popularity and empirical support [55]–[58]. The evidence that the redistribution of genetic variation through inbreeding is an important force in these community level effects is supported in studies of plant-herbivore [45], [46], [59]–[65], plant-pathogen [5]–[7], and plant-pollinator interactions [9] and this study). The loss of genetic variation in small and fragmented populations has longed been recognized as a risk factor in conservation biology [66], and Bangert *et al*. [67] have argued recently that maintaining genetic diversity in plant populations can have important conservation benefits for the communities of arthropods that depend on them. Although their study dealt specifically with the effects of host hybrid zones on insect herbivore communities, our study suggests that the same might also be true for the effects of inbreeding on pollinators. If inbreeding commonly degrades reward resources, a wide range of pollen-feeding insects (e.g., bees, beetles, thrips, and flies), birds, and mammals [43] can be impacted.

## Supporting Information

Data S1 (XLSX)
